# Membrane protein isolation and structure determination in cell-derived membrane vesicles

**DOI:** 10.1073/pnas.2302325120

**Published:** 2023-04-25

**Authors:** Xiao Tao, Chen Zhao, Roderick MacKinnon

**Affiliations:** ^a^Laboratory of Molecular Neurobiology and Biophysics, The Rockefeller University, New York, NY 10065; ^b^HHMI, The Rockefeller University, New York, NY 10065

**Keywords:** cell membrane vesicle, proteoliposome, cryo-EM, membrane protein, ion channel

## Abstract

Integral membrane protein structure determination traditionally requires extraction from cell membranes using detergents or polymers. Here, we describe the isolation and structure determination of proteins in membrane vesicles derived directly from cells. Structures of the ion channel Slo1 from total cell membranes and from cell plasma membranes were determined at 3.8 Å and 2.7 Å resolution, respectively. The plasma membrane environment stabilizes Slo1, revealing an alteration of global helical packing, polar lipid, and cholesterol interactions that stabilize previously unresolved regions of the channel and an additional ion binding site in the Ca^2+^ regulatory domain. The two methods presented enable structural analysis of both internal and plasma membrane proteins without disrupting weakly interacting proteins, lipids, and cofactors that are essential to biological function.

## Significance

Structural studies of membrane proteins historically have relied almost exclusively on first removing membrane proteins from the membrane with dispersive agents like detergents. But dispersive agents destabilize many membrane proteins, and in all membrane proteins, they remove weakly associated lipids, cofactors, and proteins essential to normal biological function. The procedures presented in this paper allow structure determination at atomic resolution without ever removing the proteins from their membrane environment. Thus, the structures of proteins that heretofore could not be extracted stably from the membrane can now be analyzed, and weakly associated lipid molecules, cofactors, and proteins normally lost in isolation are retained. These methods have immediately become the approach of choice for membrane protein structure determination in our laboratory.

Historically, the study of integral membrane protein structures has followed the development of methods to stabilize membrane proteins outside the membrane. For more than 40 years, detergents have been the mainstay approach (1–3). More recently, methods aimed to better simulate the membrane environment have been developed, including lipid cubic phase crystallization (4), bicelles (5), lipid nanodiscs (6–9), and styrene maleic acid lipid particles (10).

Very recently, several structures were determined after isolating membrane proteins in detergent and then reconstituting them back into lipid vesicles (11–19). While reconstitution of membrane proteins into lipid vesicles is a valuable approach for structural studies, the procedure is very difficult in some cases, especially if the membrane protein requires stabilization in a low critical micelle concentration detergent. Moreover, lipids used in the reconstitution will not likely replicate the composition of a cell membrane before the membrane composition is known. Of equal importance, the detergent extraction step prior to reconstitution will most assuredly result in the loss of cofactors and associated proteins that may be weakly bound yet necessary for normal biological function.

Ideally, we wish to see the structures of biological molecules in the cellular environment. To this aim, techniques such as cryoelectron tomography (20) combined with specialized sample preparation methods, e.g., focused ion beam milling (21), are being developed. To complement this strategy, we wondered whether it would be possible to isolate fragments of native cell membranes in the form of small vesicles for structural analysis at atomic resolution. To this end, we have developed two separate procedures, total membrane and plasma membrane, which, for exemplar purposes, we apply to the structure determination of a potassium channel in cell membranes, with an immediate return in the depth of our understanding of this potassium channel.

## Results

### Enrichment of Slo1-Containing Vesicles Derived from the Total Membrane.

**Fig. 1.**
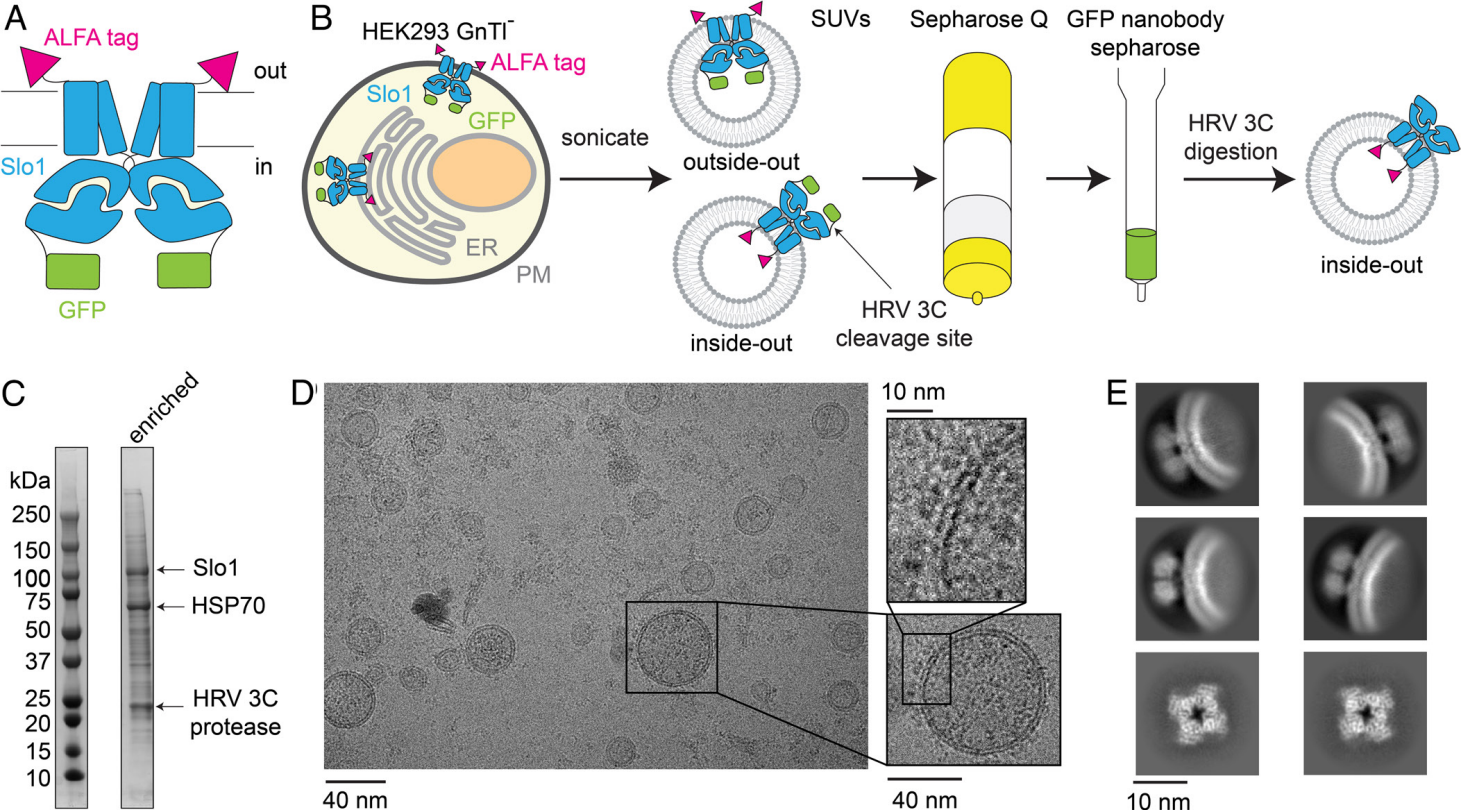
Preparation of Slo1-containing vesicles from the total cell membrane. (*A*) Construct design of the human Slo1 channel. The extracellular ALFA tag is colored in magenta, and the intracellular GFP tag is colored in green. (*B*) Purification procedure of Slo1-containing vesicles from the total membrane. The endoplasmic reticulum (ER) membrane is colored in light gray, and the plasma membrane (PM) is colored in dark gray. (*C*) SDS-PAGE of the total membrane vesicles enriched with Slo1. (*D*) Representative micrograph of Slo1-containing vesicles from the total membrane. (*E*) 2D class averages of Slo1 channel from the total cell membrane.

For this study, we used a mammalian Slo1 (high-conductance Ca^2+^-activated K^+^ channel) channel derived from Homo sapiens (22), engineered at the DNA level to contain an extracellular ALFA tag (23) at its N terminus and an intracellular green fluorescent protein (GFP) tag at its C terminus (Fig. 1*A*). This channel, which is functional when expressed in cells (*SI Appendix*, Fig. S1), permitted the enrichment of vesicles containing Slo1 in both intracellular-side-out (inside-out) and extracellular-side-out (outside-out) orientations (Fig. 1*B*). To produce cell membrane–derived vesicles containing Slo1, the ALFA-Slo1-GFP construct was heterologously expressed in HEK293 GnTl^-^ cells from suspension cultures using the BacMam method (24).

To isolate Slo1-containing vesicles from the total cell membrane fraction, cells were first disrupted by sonication into small unilamellar vesicles (SUVs), likely exhibiting both inside-out and outside-out orientations (Fig. 1*B*). These vesicles originate mainly from intracellular membranes such as the endoplasmic reticulum (ER) and the Golgi apparatus because the surface plasma membrane contributes less than 10% of the total membrane in cells (Table 12-2 in ref. 25). We then removed nucleic acids using anion exchange chromatography and enriched Slo1-containing vesicles using a resin conjugated to a GFP nanobody (Fig. 1*B*). A Coomassie-stained, denaturing gel of the final sample showed that Slo1 was highly enriched, with only two other major contaminating proteins; 70 kDa heat shock protein (HSP70) and the protease used to elute bound vesicles from the resin (Fig. 1*C*). Note that this enrichment captures only vesicles containing Slo1 with an inside-out orientation (Fig. 1 *D* and *E*). The size range of these vesicles was typically 20 to 100 nm diameter. After selecting particles using Topaz (26) with a model trained on manually picked particles, we sorted these particles by two-dimensional (2D) classification, which unambiguously revealed Slo1 channels (Fig. 1*E*). Additionally, the membrane curvatures from the 2D class averages (Fig. 1*E*) were consistent with our purification protocol that enriched Slo1 channels with an inside-out orientation (Fig. 1*B*).

### Structural Analysis of Slo1 from Total Membrane Vesicles.

**Fig. 2.**
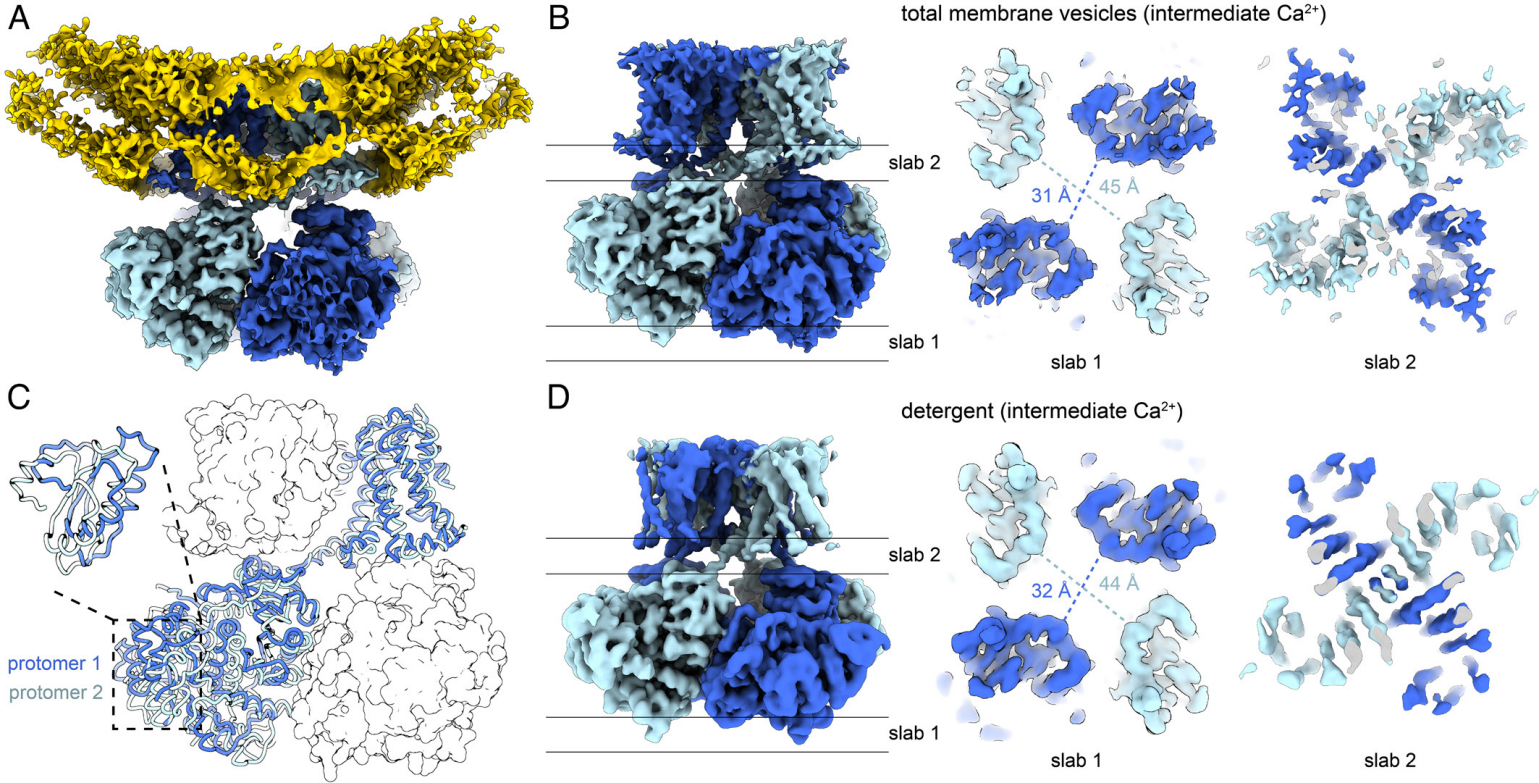
Structural analysis of Slo1 from the total membrane vesicles. (*A*) Overall cryo-EM map of Slo1 from the total membrane vesicles. The protomers in Slo1 are colored in dark and light blue. The lipid bilayer (yellow) is contoured at a low threshold to show the curvature of the membrane. (*B*) Two-fold symmetry of Slo1 from the total membrane vesicles. Slabs of cryo-EM density parallel to the membrane plane are taken at the intracellular-most end of the Slo1 gating ring (slab 1) and the pore region (slab 2). (*C*) Structural comparison of the two protomers from neighboring subunits. The structures are superposed according to the selectivity filter and the pore helix. (*D*) Two-fold symmetric structure of Slo1 determined in detergent using the same EDTA-free buffer. The slab of cryo-EM density at the same region in *B* is shown. The density at the center of the pore entryway in slab 2 is likely due to detergent molecules.

We determined the cryoelectron microscopy (cryo-EM) structure of the Slo1 channel in vesicles from the total cell membrane preparation at a resolution of 3.8 Å (Fig. 2*A* and *SI Appendix*, Figs. S2 and S3*A* and Table S1). The structure exhibited two-fold symmetry, contrasting the four-fold symmetric structures that we previously determined with detergent-solubilized Slo1 in both Ca^2+^-free and Ca^2+^-bound conformations (22, 27, 28). The two-fold symmetric organization is most evident within the cytoplasmic gating ring and is most easily appreciated when viewing the Slo1 channel along its central axis from the intracellular side. At its most intracellular extent, the intersubunit distance for two pairs of diagonally associated protomers differs by more than 10 Å (Fig. 2*B*). However, at the level of the inner membrane leaflet, the channel becomes more four-fold symmetric, and near the center of the membrane and selectivity filter, the channel follows four-fold symmetry (Fig. 2*B*). Because of the breakdown of four-fold symmetry on the intracellular side of the channel, if the two-fold symmetric channel is superposed onto its four-fold symmetric counterpart by aligning their selectivity filters, one pair of diagonally associated cytoplasmic domains is rotated and resides closer to the membrane than the other (Fig. 2*C*). A similar two-fold symmetric organization was also reported when detergent-purified Slo1 was reconstituted into lipid vesicles (18).

We wondered whether the two-fold symmetry is a consequence of the presence of a membrane or the concentrations of Ca^2+^ used when isolating vesicles, which was intermediate between the low Ca^2+^ (containing ethylenediaminetetraacetic acid (EDTA) or ethylene glycol-bis(β-aminoethyl ether)-N,N,N′,N′-tetraacetic acid (EGTA)) or high Ca^2+^ (10 mM added Ca^2+^) concentrations used when purifying Slo1 in detergent (22, 27, 28). To address this issue, we purified Slo1 in detergent (22) using the same intermediate Ca^2+^ concentration used in the total membrane vesicle preparation, i.e., no EDTA or EGTA and no additional Ca^2+^. Solving the structure, we observed two-fold symmetry, indicating that the two-fold symmetric structure is a function of the Ca^2+^ concentration, not the presence of a membrane (Fig. 2*D*). As a point separate from the main aim of the present paper, the symmetry result indicates that while Slo1 adopts predominantly four-fold symmetric structures in the absence or in the presence of high levels of Ca^2+^ (closed and open, respectively), intermediate levels of Ca^2+^ favor breakdown of this symmetry. This finding implies that gating transitions between closed and open conformations can occur through conformational changes within individual subunits that are, at least to some degree, uncoupled from each other. Such breaking of four-fold symmetry has also been observed in other members of the tetrameric cation channel family (29–35).

### Enrichment of Slo1-Containing Vesicles Derived from the Plasma Membrane.

**Fig. 3.**
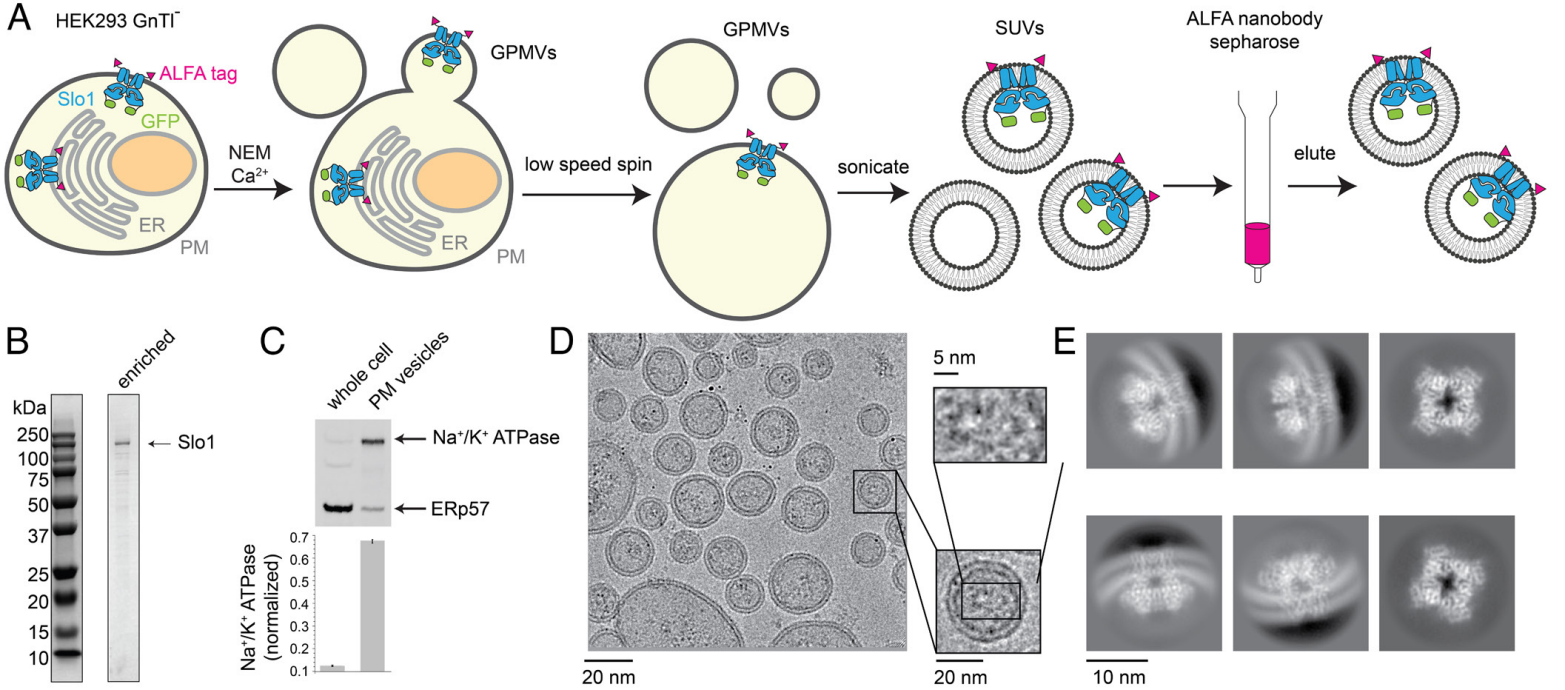
Preparation of Slo1-containing vesicles from the plasma membrane. (*A*) Purification procedure of Slo1-containing vesicles from the plasma membrane (PM). The endoplasmic reticulum (ER) membrane is colored in light gray, and the plasma membrane is colored in dark gray. (*B*) SDS-PAGE of the PM vesicles enriched with Slo1. (*C*) Western blot showing the relative abundance of the PM marker (Na^+^/K^+^ ATPase) and the ER marker (ERp57). Normalized PM marker levels for the two different samples are shown as bar graph, beneath (n = 3). (*D*) Representative micrograph of Slo1-containing vesicles from the plasma membrane. (*E*) 2D class averages of the Slo1 channel from the plasma membrane vesicles.

Many integral membrane proteins require full maturation to reach the plasma membrane in a fully functional state. To isolate Slo1-containing plasma membrane vesicles from the total cell membrane, we took advantage of the discovery of chemically induced giant plasma membrane vesicles (GPMVs) (36). GPMVs were induced from live HEK293 GnTl^−^ cells expressing ALFA-Slo1-GFP by N-ethylmaleimide (NEM) treatment in the presence of Ca^2+^ (Fig. 3*A*). These GPMVs were then sonicated to SUVs that were further enriched by ALFA-nanobody affinity resin (Fig. 3*A*). In contrast to the total membrane vesicle isolation method, this ALFA-nanobody enrichment method will isolate the Slo1-containing vesicles with an outside-out orientation. In fact, this is the orientation for the majority of the GPMV-derived SUVs, as GFP nanobody resin that interacts with the intracellular GFP tag (Fig. 1*A*) fails to enrich Slo1-containing vesicles. This outcome is consistent with the literature indicating that GPMVs tend to maintain the outside-out orientation of membrane proteins (37, 38).

The Coomassie-stained denaturing gel of the final sample contained only one major band corresponding to Slo1 (Fig. 3*B*). Western blot analysis showed that the plasma membrane marker, Na^+^/K^+^ ATPase, was enriched in the GPMVs compared to the ER marker ERp57, in contrast to the high level of the ER marker in the whole cell (Fig. 3*C*). Cryo-EM micrographs of the plasma membrane vesicles have a clean background with the top or bottom views of Slo1 readily identifiable (Fig. 3*D*). As in the total membrane method, we selected particles using Topaz and performed a 2D classification, which again unambiguously revealed the presence of the Slo1 channel (Fig. 3*E*). The membrane curvatures of the 2D classes indicate that the Slo1 channels almost exclusively adopt an outside-out orientation (Fig. 3*E*), consistent with our purification protocol.

### Structural Analysis of Slo1 from Plasma Membrane Vesicles.

**Fig. 4.**
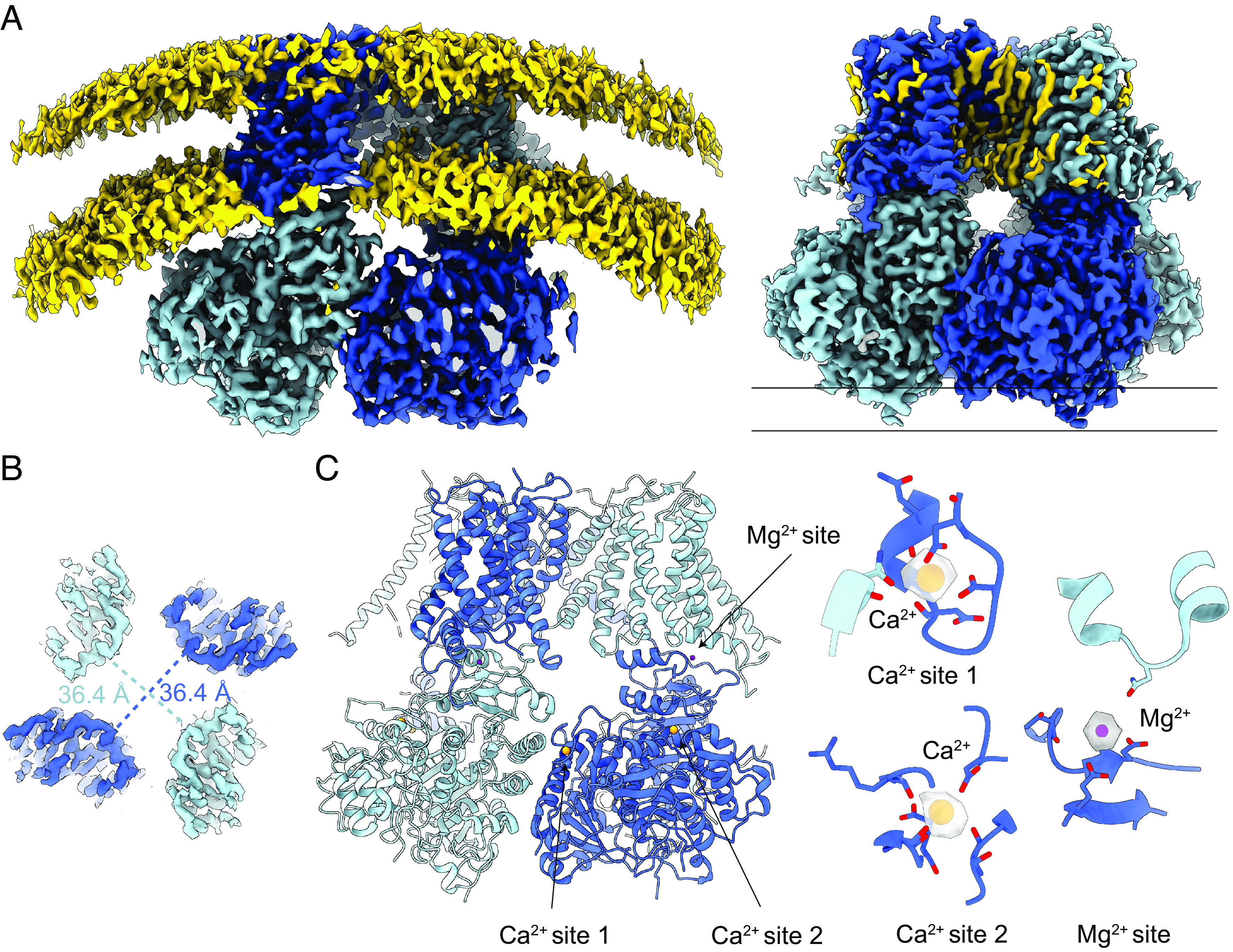
Structural analysis of Slo1 from the plasma membrane vesicles. (*A*) Overall cryo-EM map of Slo1 from the total membrane vesicles. The protomers in Slo1 are colored in dark and light blue. The lipid bilayer (yellow) is contoured at a low threshold to show the curvature of the membrane. (*B*) Four-fold symmetry of Slo1 from the plasma membrane vesicles. Slabs of cryo-EM density parallel to the membrane plane are taken at the intracellular-most end of the Slo1 gating ring indicated in *A*. (*C*) The two Ca^2+^-binding sites and the Mg^2+^-binding sites are occupied. The Ca^2+^ ion is colored in orange, and the Mg^2+^ ion is colored in purple.

We determined the cryo-EM structure of the Slo1 channel in plasma membrane-derived vesicles at a resolution of 2.7 Å (Fig. 4*A* and *SI Appendix*, Figs. S3*B* and S4 and Table S1). The high quality of the cryo-EM map resolved the transmembrane domain at a higher resolution than previously published structures of Slo1 in detergent or in reconstituted lipid vesicles (18, 22, 27, 28). Regions of the channel not resolved in our past work (22, 27, 28) appear to have been stabilized by the plasma membrane environment. Some of these will be discussed in a separate section below.

The two best-defined Slo1 structural classes from the plasma membrane vesicles are four-fold symmetric (C4), consistent with the higher Ca^2+^ concentration (Fig. 4 and *SI Appendix*, Fig. S4). These are essentially identical to each other and called the C4 structure. Two additional classes exhibit two-fold symmetry and define a C2 structure, which is distinct from the C2 structure observed in the intermediate Ca^2+^ concentrations (*SI Appendix*, Figs. S4 and S5). In the following, we focus our description on the higher resolution C4 structure in plasma membrane vesicles (Fig. 4). Both high-affinity Ca^2+^-binding sites per subunit in the gating ring are occupied due to the presence of 2 mM Ca^2+^ in the solution used to induce GPMV formation (Fig. 4*C*). The Mg^2+^ site is also occupied, likely because the normal intracellular Mg^2+^ concentration is in the millimolar range (Fig. 4*C*). Consistent with full occupation of the two Ca^2+^ and one Mg^2+^ ion-binding site per subunit, the gating ring engages the transmembrane domain as in previously determined Ca^2+^- and Mg^2+^-bound structures of Slo1 in detergent (Fig. 4*C*) (22, 27, 28), and the pore’s gate is wide open, establishing an active state of the Slo1 channel in a cell membrane. This structure, together with the demonstration that ion channels are functional in GPMVs (39), supports the suitability of GPMVs for the structural biology of membrane proteins.

### Properties of the Slo1 Channel Revealed in Plasma Membrane Vesicles.

**Fig. 5.**
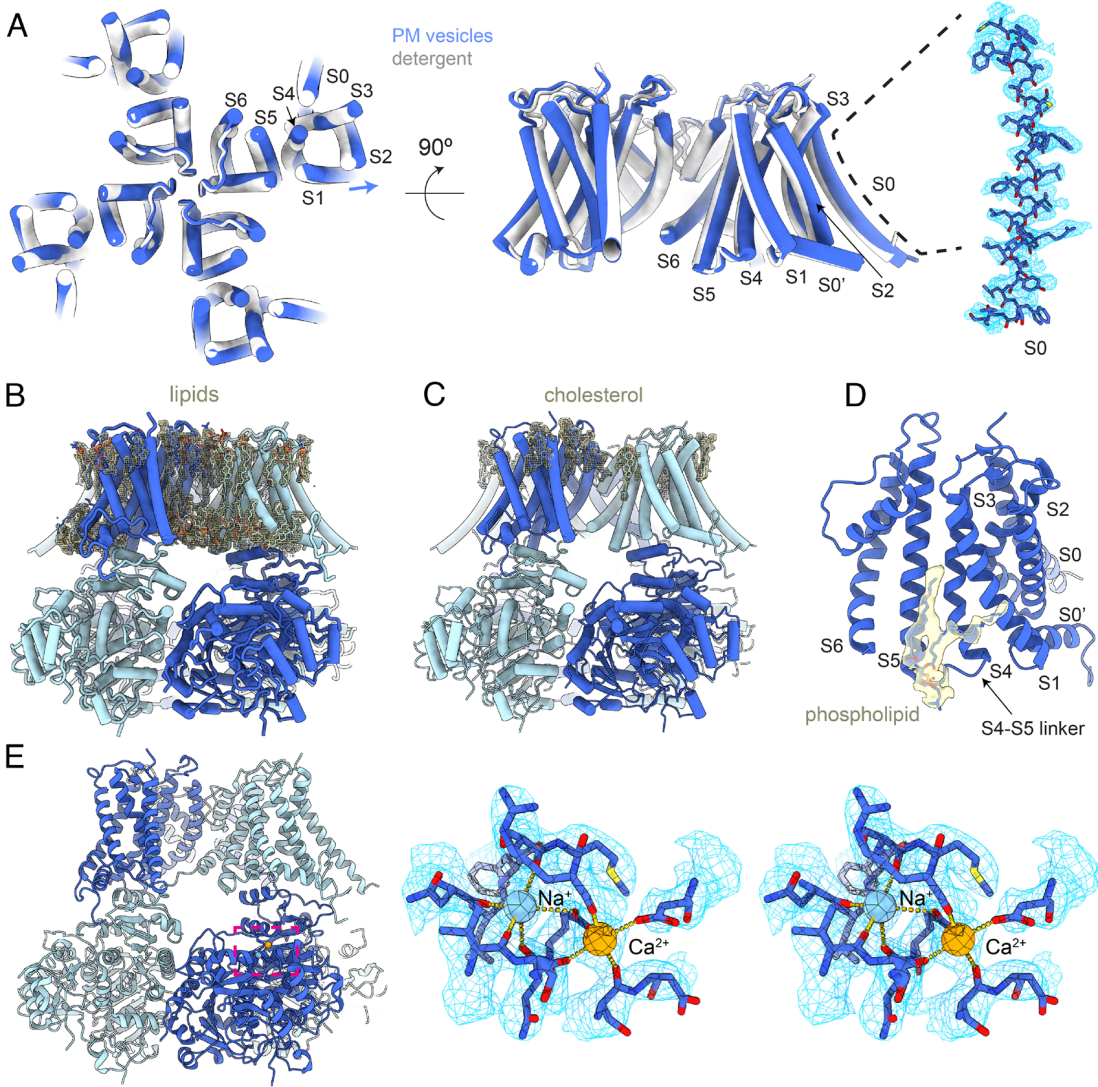
New structural features of Slo1 observed in plasma membrane vesicles. (*A*) Global conformational change of the Slo1 transmembrane domain. The models are superposed according to the transmembrane domain. The Slo1 model determined in plasma membrane (PM) vesicles is colored in blue, and the Slo1 model determined in detergent is colored in gray. A magnified view of the S0 helix and its cryo-EM density are shown on the *Right*. (*B*) Lipid (phospholipid and cholesterol) molecules observed in the cryo-EM map. The Slo1 model is colored in two shades of blue, and the cryo-EM densities for the lipid molecules are colored in light yellow. (*C*) Cholesterol molecules identified in the cryo-EM map. Cryo-EM densities for putative cholesterol molecules are colored in light yellow. (*D*) Phospholipid-binding site at the S4-S5 linker. The cryo-EM density for the phospholipid is colored in light yellow. (*E*) New cation (likely Na^+^) binding site identified in the Slo1 structure from plasma membrane vesicles. The Na^+^ ion is colored in light blue, and the Ca^2+^ ion is colored in orange. Right side is a close-up view of the magenta box on the left.The atomic model and cryo-EM density map are shown as wall-eyed stereoscopic images.

The plasma membrane environment has provided a structure of Slo1 that exhibits a much higher degree of order throughout the transmembrane region, enabling clear definition of regions that were poorly resolved in the previous cryo-EM structures in detergent (22, 27, 28) (*SI Appendix*, Fig. S6). Notably, helices on the perimeter such as the S0 helix, which makes little contact with the rest of Slo1 and was barely resolved in structures with detergent (22, 27, 28), are highly ordered (Fig. 5 *A*, *Right* and *SI Appendix*, Fig. S6*A*). This higher degree of order may be a consequence of the chemical composition and physical properties of the cell plasma membrane. Quite unexpectedly, we also observe a global repositioning of the transmembrane helices in the plasma membrane compared to detergent micelles. Fig. 5*A* shows a superposition of the transmembrane helices: While a small rotation is evident, an unmistakable expansion of the helices away from the central axis of the channel caught our attention. This does not involve the selectivity filter, nor the pore helices, which are essentially identically positioned in the two structures, but other helices are expanded away from the center. We think that it is unlikely that the curvature of the vesicle membrane caused this because the expansion is similar in both the outer and inner membrane leaflets. We suspect that the expansion, together with the higher degree of order, results from interactions of the channel with its plasma membrane environment.

A myriad of lipid molecules can be observed surrounding and within the transmembrane domain that were absent in the detergent structures (Fig. 5*B* and Movie S1). These lipids include both phospholipids and cholesterol. Compositional asymmetry with a preponderance of cholesterol in the outer membrane leaflet is evident. Indeed, a considerable fraction of the membrane-facing surface of Slo1 in contact with the outer leaflet is plastered with cholesterol molecules (Fig. 5*C*). This observation is consistent with a study showing up to a 12-fold higher concentration of cholesterol in the outer membrane leaflet of some mammalian cells (40). It thus appears that the membranes of GPMVs reflect cell plasma membranes in many respects. Numerous phospholipids make specific interactions with Slo1. We point out one phospholipid in particular because of its proximity to the S4-S5 linker, which couples the voltage sensor to the cytoplasmic Ca^2+^ sensor (Fig. 5*D*). Its headgroup interacts directly with the linker, and its alkyl chain runs into the membrane parallel to and contacting the S5 helix.

Outside the membrane in the cytoplasmic Ca^2+^ sensor, we have discovered a previously undetected cation binding site (Fig. 5*E*). This binding site is adjacent to the known Ca^2+^ site 2 (Fig. 4*C*), with a center-to-center distance between ions about 5.4 Å. The Ca^2+^ is coordinated by oxygen atoms from two carboxylate side chains and three main chain carbonyl oxygen atoms. The new cation is coordinated by four main chain carbonyl oxygen atoms (Fig. 5*E*). The nearest carboxylate oxygen (coordinating the neighboring Ca^2+^) is about 3.5 Å away. The ion-oxygen distances for the carbonyl oxygen atoms as well as the coordination number are compatible with the new cation being either Ca^2+^ or Na^+^; however, given the net charge of the coordinating cage, the newly discovered ion is likely to be Na^+^ (41). Na^+^ is known to regulate the Slo2 channel (42–44), a close relative of Slo1. It will be interesting to examine the role of this new ion-binding site in the function of Slo1.

## Discussion

In this study, we showed using two different methods how to isolate a membrane protein from cells without ever removing the protein from its lipid environment and thus maintaining a more native environment. The first approach should be suitable for isolating membrane proteins that normally reside in intracellular membranes, especially—but not limited to—the ER and Golgi. The second approach permits isolation of plasma membrane proteins derived from the plasma membrane and is thus important for the study of fully mature plasma membrane proteins and their interactions with surrounding lipids and other proteins. Native lipid vesicles from cells were used decades ago for electrophysiology studies (45–48) and more recently for native mass spectrometry of protein complexes (49). Here, we enriched these cell-derived vesicles containing a specific membrane protein and determined its high-resolution structures using cryo-EM.

The resolutions of the protein structures from cell-derived membranes are comparable to those we have solved using detergent micelles. In fact, the structure of Slo1 from GPMVs is better defined in regions on the perimeter of the protein such as the S0 transmembrane helix. We assume that these improved regions were somewhat disordered in earlier studies because of the dispersive nature of the detergent environment. Perhaps owing to the stabilization provided by a plasma membrane-like environment, we also discovered new features of Slo1, including some structural components not previously resolved, specific interactions with membrane phospholipids and cholesterol, and a new cation-binding site in the Ca^2+^ sensor.

The advantages to cell-derived membrane vesicles are multiple. 1) They eliminate the need to remove a protein from the membrane, which is essential for proteins that are unstable outside the membrane. 2) They preserve a local environment for the protein that is closer to its natural environment in the cell. 3) Combined, the two isolation methods should permit the analysis of proteins resident in internal membranes, in plasma membranes, and plasma membrane proteins along their pathway of assembly and maturation. 4) It seems likely that associated molecular partners such as small molecules, lipids, and other proteins that normally dissociate under traditional purification methods will be discovered by analyzing samples prepared by the described methods. In other words, the methods should enable the structural study of transient membrane protein complexes.

We enumerate several potential limitations to structural analysis with cell-derived membrane vesicles. 1) Native proteins that may exist in membranes at low abundance will be more challenging. The Slo1 channel, even with heterologous expression, did not express to very high levels, and thus enrichment using an affinity tag was important. With gene modification, native proteins can be similarly tagged and enriched. Further experience will be needed before we know how well these methods apply to proteins present at low density. 2) Slo1, with its large cytoplasmic structure, was easily identifiable and amenable to single-particle cryo-EM analysis. Smaller, more membrane-embedded proteins like certain G-protein-coupled receptors, for example, will be more challenging. In these more challenging cases, fiducials such as antibody fragments directed against the target protein ought to help (50, 51). 3) Because thin ice is a requirement for cryo-EM, the lipid vesicles must be made sufficiently small to fit into the thin ice layer. Of course, this same constraint also applies when studying membrane proteins reconstituted into lipid vesicles. Small vesicles have high membrane curvature, and therefore, a sizeable bending force is applied to proteins embedded in them (15). In our experience with five different K^+^ channels reconstituted into lipid vesicles, the structures are very similar to those observed in detergent micelles (except when membrane voltage is applied across the membrane). In Piezo1, a mechanosensitive channel that naturally changes its shape in response to bending forces, vesicle size modulates its shape (14). From these observations, we think that most membrane proteins are relatively shape-insensitive to the bending forces applied to them in small vesicles; however, it is likely that in some cases, the bending forces will alter a protein’s structure. This possibility should be kept in mind when using these methods. 4) The fourth "limitation" is more of an uncertainty, but of great importance. To what extent are GPMVs like plasma membranes? Although previous studies reported some preliminary analyses (52, 53), there is much to be learned about the composition and organization of GPMVs produced by the method we used. This is a subject of intense study in our laboratory.

In summary, the methods presented here offer a less disruptive approach to the structure determination of membrane proteins. We anticipate that they will accelerate the study of membrane and membrane-associated proteins, especially by permitting the isolation of transient complexes.

## Materials and Methods

### Cell Culture.

*Spodoptera frugiperda* Sf9 cells (ATCC CRL-1711) were cultured in Sf-900 II SFM medium supplemented with 100 U/mL penicillin and 100 U/mL streptomycin at 27 °C. HEK293S GnTl^−^ cells (ATCC CRL-3022) were cultured in Freestyle 293 medium supplemented with 2% fetal bovine serum (FBS), 100 U/mL penicillin, and 100 U/mL streptomycin at 37 °C. PtK2 cells (ATCC CCL-56) were cultured in ATCC-formulated Eagle's Minimum Essential Medium (ATCC 30-2003) supplemented with 10% FBS.

### Construct Design.

The DNA sequence encoding ALFA peptide (23) was inserted into the N terminus of the human Slo1 construct used in the previous cryo-EM study (22). The resulting protein has the green fluorescent protein (GFP) followed by a 1D4 antibody recognition sequence (TETSQVAPA) on the C terminus, separated by an human rhinovirus (HRV)-3C protease cleavage site (SNSLEVLFQ/GP), as well as the ALFA tag at the N terminus.

### Protein Expression.

Heterologous expression of Slo1 was carried out using the BacMam method as previously described (22, 24). In brief, bacmids were generated by transforming the ALFA-Slo1-HRV3C-GFP-1D4 construct into *Escherichia coli* DH10Bac cells. Baculoviruses were then produced by transfecting Sf9 cells with the bacmid using Cellfectin II (Invitrogen).

Baculoviruses, after two rounds of amplification, were used for cell transduction. Suspension cultures of HEK293S GnTI^-^ cells were grown at 37 °C to a density of ~3 × 10^6^ cells/mL and infected with 10% (v:v) of the baculovirus. After 20 h, 10 mM sodium butyrate was supplemented, and the temperature was shifted to 30 °C. Cells were harvested ~40 h after the temperature switch. For total membrane isolation, cell pellets were flash-frozen in liquid N_2_ and used later. For plasma membrane isolation, cell pellets were used immediately without freezing.

### Total Membrane Vesicle Preparation.

All steps were performed at 4 °C in the cold room. Frozen pellet from 4 L cells was thawed and resuspended in 160 mL lysis buffer [20 mM K-HEPES pH 7.4, 300 mM KCl, 0.5 mM MgCl_2_, and 5 mM dithiothreitol (DTT)] supplemented with 10 µg/mL leupeptin, 10 µg/mL pepstatin A, 1 mM benzamidine, 2 μg/mL aprotinin, 0.3 mg/mL AEBSF, ~100 µg/mL DNase, and ~100 µg/mL RNase A. The cell resuspension was then homogenized in a Dounce homogenizer for ~30 to 60 strokes. The homogenate was transferred to a metal beaker and sonicated in an ice bath with a probe sonicator (1/2” tip with the Branson102-C converter) at 60% power. The homogenate was disrupted by 30 s pulses four times, with a 30 s delay in between pulses.

The sonicated homogenate was then spun at 12,000 g for 10 min. EDTA was added to the supernatant containing small vesicles, and then, the sample was spun again at 12,000 g for 10 min. We added EDTA to potentially reduce divalent ion–mediated vesicle and protein aggregation, but we have not tested whether incorporating EDTA is necessary. EDTA was not included in the previous steps because divalent ions are important for the activities of DNases and RNases. The resulting supernatant was filtered through 0.8 μm syringe filters and loaded onto a 20 mL Q Sepharose Fast Flow resin (Cytiva 17-0510-01) column preequilibrated with supplemented lysis buffer containing EDTA in a gravity column. The flow-through was collected and resin further washed with 3 column volumes (CVs) of EDTA-containing supplemented lysis buffer.

The flow-through and 3 CVs of wash buffer were combined, and 1 mM fluorinated fos-choline-8 (FFC8, Anatrace, F300F) was added to minimize nonspecific hydrophobic interactions with the GFP nanobody–conjugated affinity resin (54). The resulting sample was incubated with 4 mL GFP-nanobody resin (CNBr-activated Sepharose 4B resin from GE Healthcare) in batch at 4 °C for 1 h. The resin was loaded onto a gravity column and subsequently washed with 80 mL wash buffer A containing 20 mM K-HEPES, pH 7.4, 500 mM KCl, 5 mM DTT, 5 mM EDTA, and 1 mM FFC8 and 80 mL wash buffer B containing 20 mM K-HEPES, pH 7.4, 300 mM KCl, and 5 mM DTT. The bound vesicles were eluted by incubating with HRV-3C protease at a final concentration of ~0.014 mg/mL at 4 °C for 2 h.

The elution and ~8 mL of additional wash with wash buffer B were combined and concentrated to OD_280_ ~2 using an Amicon 2 mL concentrator (molecular weight cutoff of 100 kDa).

The sample purity was evaluated by sodium dodecyl sulfate–polyacrylamide gel electrophoresis (SDS-PAGE) (Bio-Rad Mini-PROTEAN® TGX™ Precast Gels 4 to 15%). In Fig. 1*C*, Slo1 was identified by residual GFP fluorescence (after HRV 3C protease cleavage) measured in gel. HSP70 was identified by mass spectrometry of a similar preparation. HRV 3C protease was identified by its size on the SDS-PAGE of the purified protease by itself.

### Plasma Membrane Vesicle Preparation.

One liter of live cells (~3 to 4 × 10^6^/mL) was harvested by centrifugation at 3,000 g for 10 min. The cell pellet was resuspended in 200 mL GPMV buffer containing 10 mM K-HEPES, pH 7.4, 140 mM NaCl, 10 mM KCl, and 2 mM CaCl_2_. The cells were centrifuged again at 3,000 g for 10 min and then resuspended in 400 mL GPMV buffer supplemented with NEM (Thermo Scientific™ Pierce™, 23030) at 7.5 mM. The cell resuspension was transferred to four 250 mL baffled flasks to contain 100 mL in each flask. The flasks were then incubated in a 37 °C incubator shaking at 130 rpm for 1.5 to 2 h. The fast shaking helps to dislodge the GPMVs from the cells, resulting in smaller unilamellar vesicles (SUVs). At the end of the incubation, the flasks were shaken by hand for ~30 s. The suspension was then spun down at 3,000 g at 4 °C for 10 min to remove cells and large GPMVs.

Next, the supernatant was supplemented with 10% glycerol to prevent aggregation and sonicated with a probe sonicator (Branson, 1/2” tip with Branson102-C converter) at 40% power for three 30 s pulses with ~1 min chilling on ice in between pulses. The vesicles were then pelleted by ultracentrifugation at 100,000 g at 4 °C for 40 min in a Ti70 rotor.

The membrane vesicle pellet from every ~25 mL sample was triturated in ~1 mL GPMV buffer supplemented with 10% glycerol by gently squirting the buffer toward the pellet (with a 200 μL tip). The resuspended vesicles in the ultracentrifugation tubes were then sonicated in a water bath sonicator (Branson M1800) with ~10 s pulses at room temperature until the solution was opalescent (generally within 30 to 60 s). The vesicles were then centrifuged at 3,500 g at 4 °C for 10 min to remove the remaining aggregates. The supernatant (~20 mL) was incubated with 1 mL ALFA Selector CE resin (NanoTag) preequilibrated with GPMV buffer supplemented with 10% glycerol at 4 °C overnight.

The following day, the ALFA Selector CE resin with bound vesicles was first batch-washed twice with ~20 mL glycerol-supplemented GPMV buffer and spun at 1,000 g at 4 °C for 1 min to collect resin, followed by a second wash with 15 mL GPMV buffer supplemented with 10% glycerol. The resin was then loaded onto a gravity column and washed further with another 5 mL GPMV buffer containing glycerol, followed by 15 mL GPMV buffer without glycerol. The vesicles were then eluted 3 times with 5 CVs, 5 CVs, and 3 CVs of GPMV buffer supplemented with 0.2 mM ALFA peptide (NanoTag) by incubating at RT for 30 min. The vesicles were kept on ice after being eluted. All three elutions were then combined and concentrated to an OD_280_ ~1.5 using an Amicon 2 mL concentrator (molecular weight cutoff of 100 kDa).

### Purification of Slo1 in Detergent with Intermediate Ca^2+^.

Purification of the ALFA-tagged Slo1 protein was performed as previously described with modifications of the buffer compositions used (22). In brief, cells were gently disrupted by stirring in a hypotonic solution containing 10 mM Tris-HCl pH 8.0, 3 mM DTT, 1 mM EDTA supplemented with protease inhibitors including 0.1 μg/mL pepstatin A, 1 μg/mL leupeptin, 1 μg/mL aprotinin, 0.1 mg/mL soy trypsin inhibitor, 1 mM benzamidine, 0.1 mg/mL 4-(2-aminoethyl) benzenesulfonyl fluoride hydrochloride (AEBSF), and 1 mM phenylmethylsulfonyl fluoride (PMSF). Cell lysate was then centrifuged for 30 min at 30,000 g, and then, the pellet was homogenized by a Dounce homogenizer in a buffer containing 20 mM Tris-HCl, pH 8.0, 320 mM KCl supplemented with protease inhibitors including 0.1 μg/mL pepstatin A, 1 μg/mL leupeptin, 1 μg/mL aprotinin, 0.1 mg/mL soy trypsin inhibitor, 1 mM benzamidine, 0.1 mg/mL AEBSF, and 0.2 mM PMSF. The lysate was extracted with 10 mM lauryl maltose neopentyl glycol and 2 mM cholesteryl hemisuccinate for an hour with stirring and then centrifuged for 40 min at 30,000 g. Supernatant was added to GFP nanobody–conjugated affinity resin (CNBr-activated Sepharose 4B resin from GE Healthcare) preequilibrated with wash buffer (20 mM K-HEPES, pH 7.4, 450 mM KCl, 0.06% digitonin (Sigma) and 0.1 mg/ml 1-palmitoyl-2-oleoyl-sn-glycero-3-phosphoethanolamine (POPE):1-palmitoyl-2-oleoyl-glycero-3-phosphocholine (POPC):1-palmitoyl-2-oleoyl-sn-glycero-3-phosphate (POPA), 5:5:1 (w:w:w). The suspension was mixed by nutating for ~2 h. Beads were first washed with 10 CVs of wash buffer in a batch mode and then collected on a column by gravity, washed with another 20 CVs of wash buffer. The protein was then digested on resin with HRV 3C protease (~20:1 w:w ratio) overnight with gentle rocking. Flow-through was then collected, concentrated, and further purified on a Superose-6 size exclusion column (10/300 GL) in 20 mM HEPES-KOH, pH 7.4, 450 mM KCl, 5mM DTT, 0.06% digitonin, and 0.05 mg/mL POPE:POPC:POPA, 5:5:1 (w:w:w). All purification procedures were carried out either on ice or at 4 °C. The peak fractions corresponding to the tetrameric Slo1 channel were concentrated to about 7.5 mg/mL using a 4 mL Amicon concentrator (molecular weight cutoff of 100 kDa) and used for preparation of cryo-EM sample grids.

### Excised Inside-Out Patch Recordings.

The function of the ALFA-Slo1-HRV3C-GFP-1D4 construct used for all the cryo-EM sample preparation was verified by measuring the Ca^2+^ sensitivity of the channel in PtK2 cells in voltage-clamp inside-out patch configuration.

Specifically, 1.2 μg of the plasmid was transfected into PtK2 cells at about 50 to 60% confluency using FuGENE HD transfection reagent following the manufacturer’s instructions (Promega). Cells were transferred to 30 °C after transfection, and recordings were carried out 18 to 24 h posttransfection.

Pipettes of borosilicate glass (Sutter Instruments; BF150-86-10) were pulled to ~2 to 3 MΩ resistance with a micropipette puller (Sutter Instruments; P-97) and polished with a microforge (Narishige; MF-83). All recordings were performed at room temperature in voltage-clamp excised inside-out patch configuration with an Axopatch 200B amplifier (Molecular Devices), Digidata 1440A analog-to-digital converter interfaced with a computer, and pClamp10.5 software (Axon Instruments, Inc) for controlling membrane voltage and data acquisition. The recorded signal was filtered at 1 kHz and sampled at 10 kHz.

The bath solution contained 20 mM Na-HEPES, 136 mM K-gluconate, 4 mM KCl, and 10 mM glucose, pH 7.4 (adjusted with NaOH), with an osmolarity of ~300 Osm/L. The bath solution supplemented with 2 mM MgCl_2_ was used as the pipette solution. Ionic current under a voltage-family protocol was measured with local perfusion of bath solution or bath solution with an additional 10 μM CaCl_2_ using a fast-pressurized microperfusion system (ALA Scientific; ALAVC3 × 8 PP). Note here that the Ca^2+^ concentration refers to the amount of Ca^2+^ added from a stock of CaCl_2_, not the free [Ca^2+^].

### Grid Preparation and Data Collection.

Quantifoil R1.2/1.3 400 mesh holey carbon gold grids were glow-discharged for 22 s. Then, 3.5 μL concentrated membrane vesicles were applied to freshly glow-discharged grids and left for 3 min at 22 °C with a humidity of 100%. The grids were then blotted manually from the edge with a piece of filter paper. Another 3.5 μL sample was applied, and the grids were blotted using a Vitrobot Mark IV with a blot force of 0 and blot time of 3 s after 20 s of incubation. The grids were then flash-frozen in liquid ethane and stored in liquid nitrogen until data collection.

The concentrated detergent sample was supplemented with 2.9 mM FFC8 immediately prior to grid preparation. Then, 3.5 μL of the mixture was applied to freshly glow-discharged Quantifoil R0.6/1 300 mesh holey carbon gold grids at 22 °C with a humidity of 100%. The grids were blotted using a Vitrobot Mark IV with a blot force of 22 and blot time of 4 s after 15 s of incubation, then flash-frozen in liquid ethane, and stored in liquid nitrogen until data collection.

The dataset for Slo1-containing total membrane vesicles was collected on a 300 keV Titan Krios transmission electron microscope equipped with a Gatan K3 Summit camera, an energy filter (slit width 20 eV), and a CS corrector. A total of 21,348 movies were collected in a superresolution mode with a physical pixel size of 1.08 Å and a target defocus value of −1.0 to −2.0 μm. Every movie has a total exposure time of 2 s separated into 40 frames with a dose rate of 30 e^−^/pixel/s, giving a total dose of 51.4 e^−^/Å^2^ (1.286 e^−^/Å^2^/frame).

The dataset for Slo1-containing plasma membrane vesicles was collected on a 300 keV Titan Krios transmission electron microscope equipped with a cold-field emission gun and an energy filter (slit width 6 eV). A total of 24,876 movies were recorded by a Falcon 4i camera with a physical pixel size of 0.743 Å and a target defocus value of −1.0 to −2.0 μm. The movies have 1,505 internal frames and a total dose of 60 e^−^/Å^2^.

The dataset for the Slo1 detergent sample was collected at a 300 keV Titan Krios transmission electron microscope equipped with a Gatan K3 Summit camera, an energy filter (slit width 20 eV), and a CS corrector. A total of 18,495 movies were collected in the superresolution mode with a physical pixel size of 1.08 Å and a target defocus value of −0.7 to −2.2 μm. Every movie has a total exposure time of 2 s separated into 40 frames with a dose rate of 30 e^−^/pixel/s, giving a total dose of 51.4 e^−^/Å^2^ (1.286 e^−^/Å^2^/frame).

### Cryo-EM Data Processing for Slo1 Total Membrane Vesicles.

The raw movies were motion-corrected by MotionCor2 (55) in Relion V3.1 (56). The CTF parameters were estimated by patch CTF estimation in cryoSPARC (V4.2.0) (57), and the initial 1,000 particles were manually picked from 164 Topaz (26) denoised micrographs. These particles were then used to train a Topaz model that was used for particle picking from all micrographs. Duplicate particles were removed, and multiple rounds of 2D classification and deep 2D classification (12) were carried out to select particles with clear Slo1 density. The initial model of Slo1 in total membrane vesicles was generated by ab initio reconstruction with C1 symmetry, and then, the particles were further sorted by heterogenous refinement.

The sorted particles were subjected to another round of 2D classification in cryoSPARC (V4.2.0). The particles belonging to classes of side views (54%), top or bottom views (14%), and tilted views (32%) were used separately to train three different Topaz models, which were then used to pick particles from all micrographs. Combined particles were then subjected to 2D classification to remove "junk" particles. The selected particles were then used for ab initio reconstruction, heterogenous refinement, and homogeneous refinement in cryoSPARC (V4.2.0). It should be noted that refinement with C2 symmetry consistently yielded better-defined maps with higher resolution than refinement with C4 symmetry.

After a best model was achieved, the particles were further sorted by a 3D classification without alignment using Relion (V4.0-beta-1), with a user-defined mask covering the transmembrane domain. The resulting 85,135 particles were further refined using local refinement with C2 symmetry to generate the final cryo-EM map.

### Cryo-EM Data Processing for Slo1-Containing Plasma Membrane Vesicles.

The raw movies in EER format were divided into 50 fractions and upsampled by a factor of 2 (8k rendering) and were motion-corrected by patch motion correction with further Fourier cropping by a factor of 2, after which the CTF parameters were estimated by patch CTF estimation in cryoSPARC V4.0.1. The initial 1,363 particles manually picked from 42 micrographs were sorted by a 2D classification. The particles belonging to classes with clear Slo1 features were used to train a Topaz model for particle picking from all micrographs.

Duplicated particles were removed, and the resulting particles were subjected to multiple rounds of 2D classification to remove false-positive and low-abundance classes. A total of 597,254 particles were selected, and an initial model was generated through ab initio reconstruction in cryoSPARC V4.0.1. Heterogenous refinement with the Slo1 initial model and 2 bad models from the ab initio reconstruction yielded a good class of Slo1 containing 433,531 particles. The selected particles were further sorted by several rounds of 2D classification to remove false-positive and suboptimal classes. Orientation and translational parameters for the resulting 330,063 particles were then refined using the homogenous refinement algorithm followed by local refinement in cryoSPARC V4.0.1 with C4 symmetry. The refined particle images were subjected to Relion’s (V4.0.0) 3D classification algorithm without a mask, skipping image alignment while imposing C2 symmetry. Four out of the 6 requested classes yielded 3D reconstructions representing Slo1 with high resolutions. Further refinement of each of these 4 good classes with the nonuniform refinement algorithm in cryoSPARC V4.0.1 while applying C1, C2, or C4 demonstrated that 2 classes obey C4 symmetry, while the other 2 appear to obey C2 symmetry. Note that the conformational differences of the 2 pairs of protomers in these plasma membrane vesicle C2 classes are much smaller than the differences observed in the C2 classes of the total membrane vesicle or detergent sample preparations with an intermediate [Ca^2+^]. Local refinement of the 2 C4 classes and 2 C2 classes from plasma membrane vesicles yielded maps with resolutions of 2.7 Å, 2.9 Å, 3.0 Å, and 3.1 Å, respectively (*SI Appendix*, Fig. S4), as assessed by Fourier shell correlation using the 0.143 cutoff.

### Cryo-EM Data Processing of Slo1 in Detergent under Intermediate [Ca^2+^].

Dose-fractionated superresolution images were 2 × 2 down-sampled by Fourier cropping for motion correction with MotionCorr2 (5 × 5 patches). The parameters of the contrast transfer function were estimated by Ctffind4 (58). Following motion correction, ~1,500 particles from a subset of the images were interactively selected using Relion to generate templates representing different views for automated particle selection with Gautomatch (59). The autopicked particles were then subjected to 2 rounds of 2D classification in cryoSPARC V4.0.1 to remove junk particles or particles belonging to low-abundance classes. An initial model was generated from the selected particles using ab initio reconstruction in cryoSPARC V4.0.1.

Orientation and translational parameters of ~1,032 k selected particle images were refined with the homogeneous refinement algorithm followed by local refinement in cryoSPARC V4.0.1 imposing C1 symmetry. The refined particle images were subjected to Relion’s (V4.0.0) 3D classification algorithm without image alignment or a mask, requesting six classes. Orientation and translational parameters for the ~123k particles in the best class were refined using homogeneous refinement, followed by nonuniform refinement and local refinement (imposing C2 symmetry, which consistently yielded better-defined maps with higher resolution than refinement with C4 symmetry, similar to the total membrane vesicle data) in cryoSPARC V4.0.1, resulting in a map at a resolution of 3.3 Å before postprocessing.

## Supplementary Material

Appendix 01 (PDF)

Movie S1. **Protein and lipid density in the cryo-EM map of hSlo1 in plasma membrane vesicles.** Protein residues are colored in white, lipid molecules in yellow, and the cryo-EM density in blue. When only the protein density is shown, the protein model is represented as sticks. When only the lipid density is shown, the protein model is rendered as a Cα trace, and the lipid molecules are represented as sticks.

## Data Availability

Cryo-EM density maps and atomic coordinates of the hSlo1 channel in total membrane vesicles (Ca^2+^-free and EDTA-free), in plasma membrane vesicles, and in digitonin (Ca^2+^-free and EDTA-free) have been deposited in the Electron Microscopy Data Bank under accession codes EMD-40038 (60), and EMD-40044 (61), EMD-40045 (62) and in the Protein Data Bank under accession codes 8GH9 (63), 8GHF (64), and 8GHG (65), respectively.
