# Supplementary material for: Membrane protein isolation and structure determination in cell-derived membrane vesicles
Source: Proc Natl Acad Sci U S A. 2023 Apr 25;120(18):e2302325120. doi: 10.1073/pnas.2302325120 (PMC10160969; doi:10.1073/pnas.2302325120)
Supplement: Supplementary file 1 — Appendix 01 (PDF) [file pnas.2302325120.sapp.pdf]

PNAS

Supporting Information for

Membrane protein isolation and structure determination in cell-derived membrane vesicles

Xiao Tao[a,b,1], Chen Zhao[a,b,1] and Roderick MacKinnon[a,b,*]

a Laboratory of Molecular Neurobiology and Biophysics, The Rockefeller University, New York, NY, 10065, United States.

b Howard Hughes Medical Institute, The Rockefeller University, New York, NY, 10065, United States.

1 X.T. and C.Z. contributed equally to this work

* Roderick MacKinnon

Email: mackinn@rockefeller.edu

This PDF file includes:

Figures S1 to S6

Table S1

Legends for Movie S1

Other supporting materials for this manuscript include the following:

Movie S1

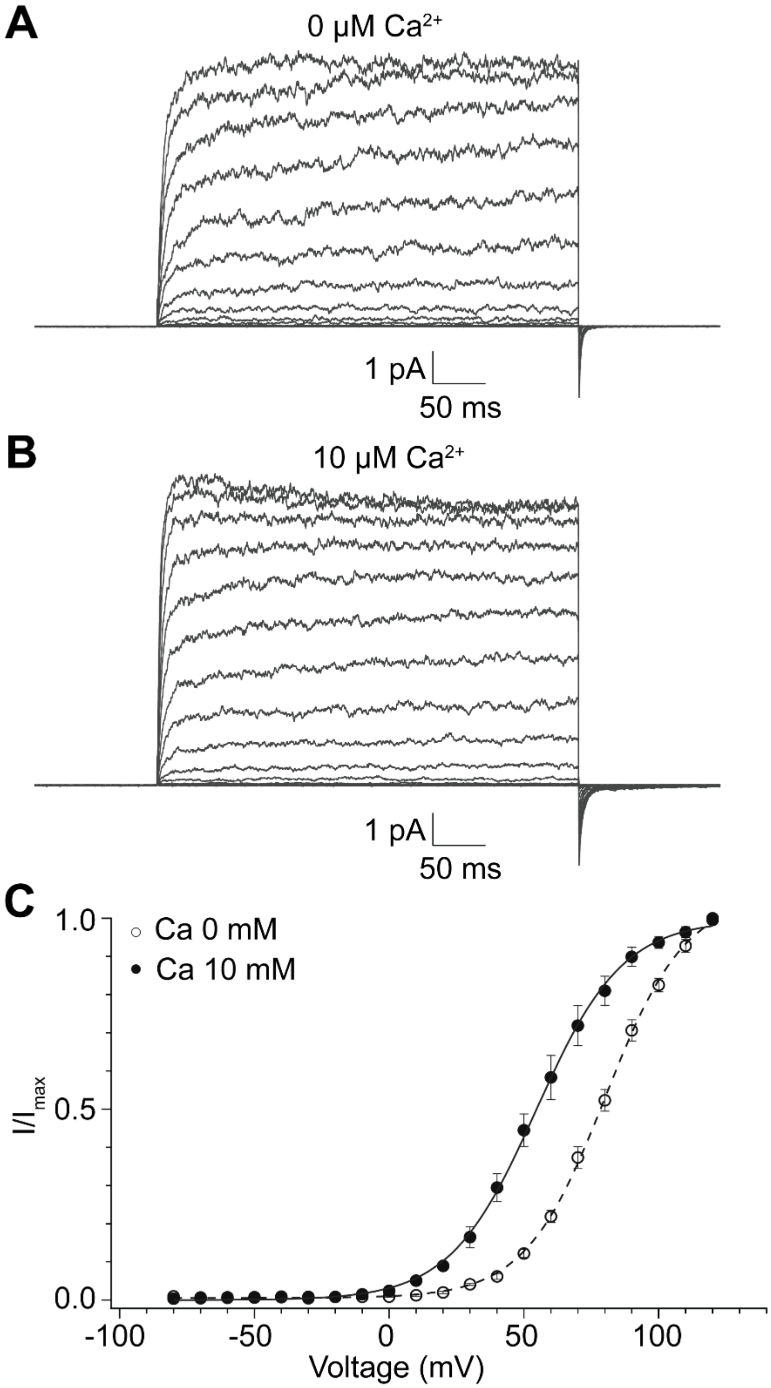

Fig. S1. Electrophysiological studies of the ALFA-Slo1-GFP in PtK2 cells. (**A, B**) Voltage-dependent channel activation of ALFA-Slo1-GFP without (**A**) and with additional 10 μM $CaCl_2$ (**B**). Representative current traces recorded using the excised inside-out patch configuration are shown. Voltage protocol: holding potential −80 mV, depolarized from −80 to +120 mV in 10 mV increment steps, stepping back to −20 mV. (**C**) The voltage-activation curve of ALFA-Slo1-GFP at 0 and 10 μM (added) $CaCl_2$. At each Ca^{2+} concentration, fraction of the maximum tail current at that concentration (n = 5; means ± SEM) is plotted against the depolarization voltage and fitted with a two-state Boltzmann function.

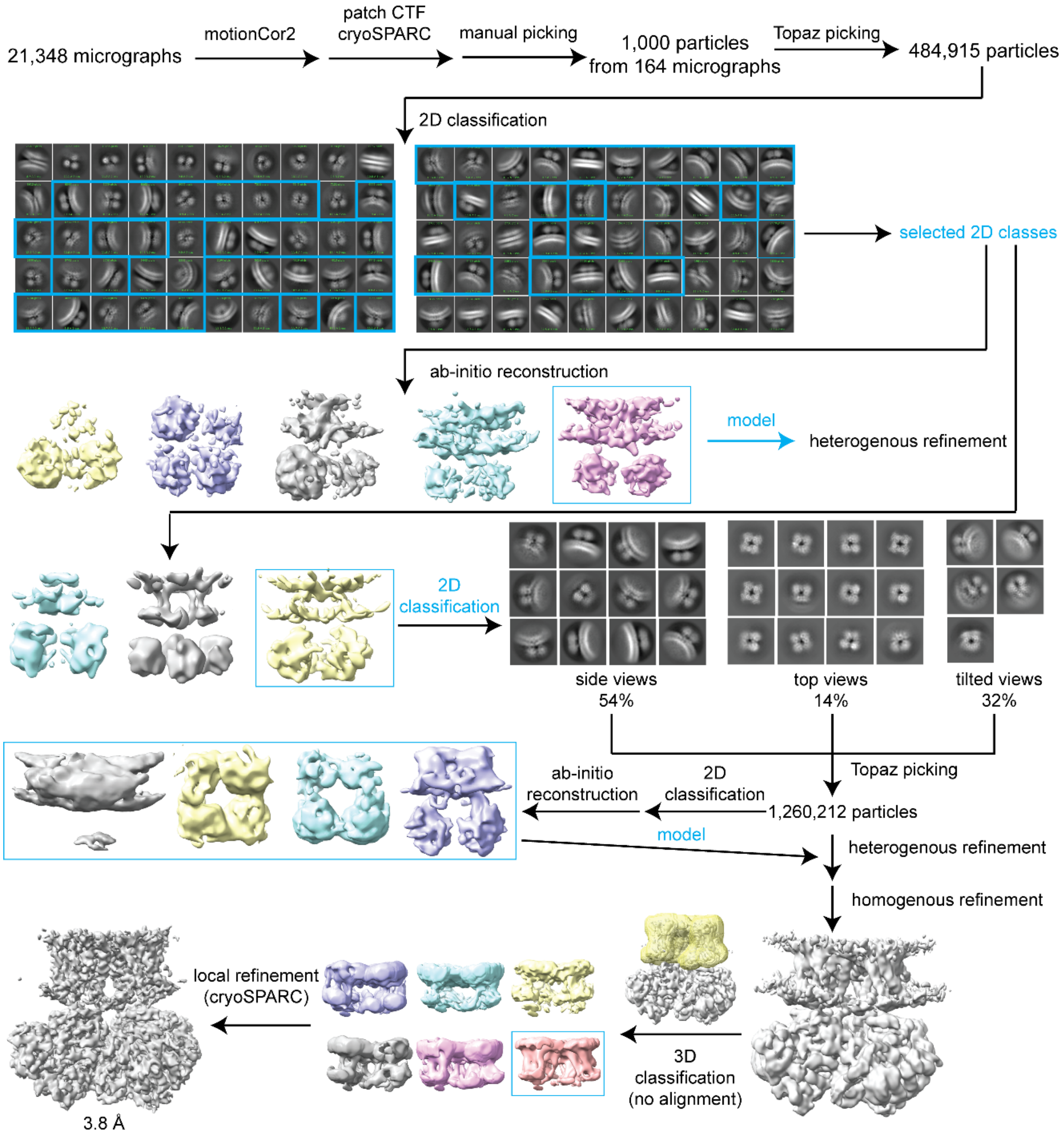

Fig. S2. Cryo-EM data processing procedure for hSlo1 in total membrane vesicles.

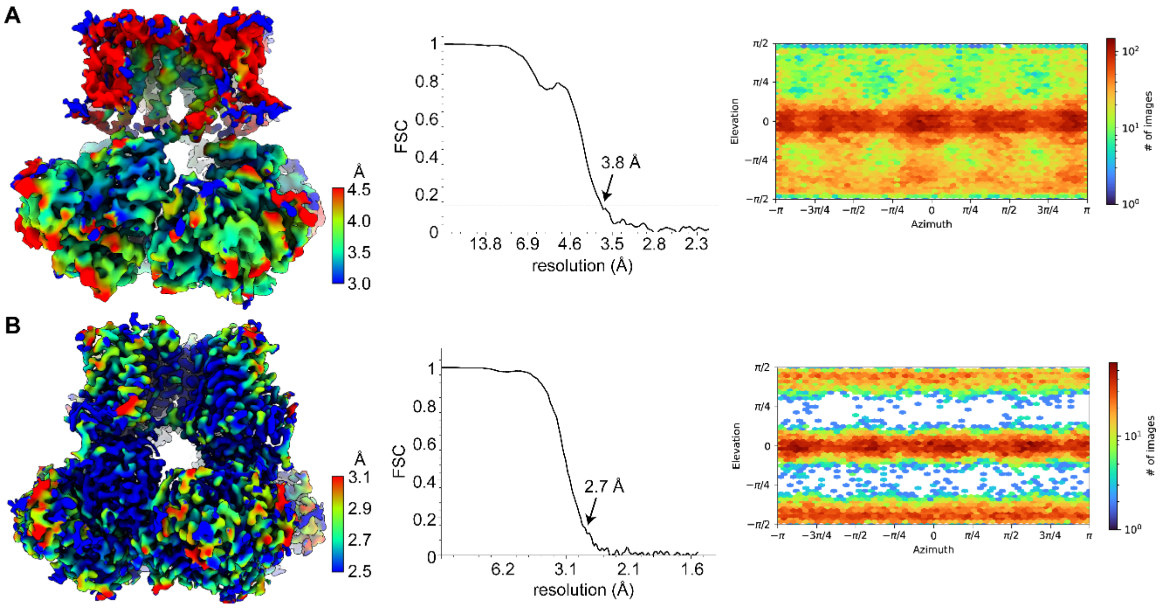

Fig. S3. Local resolution maps, FSC curves and angular distributions of hSlo1 in cell-membrane-derived vesicles. The local resolution maps and FSC curves are generated by CryoSPARC. (**A**) hSlo1 in total membrane vesicles. (**B**) hSlo1 in plasma membrane vesicles.

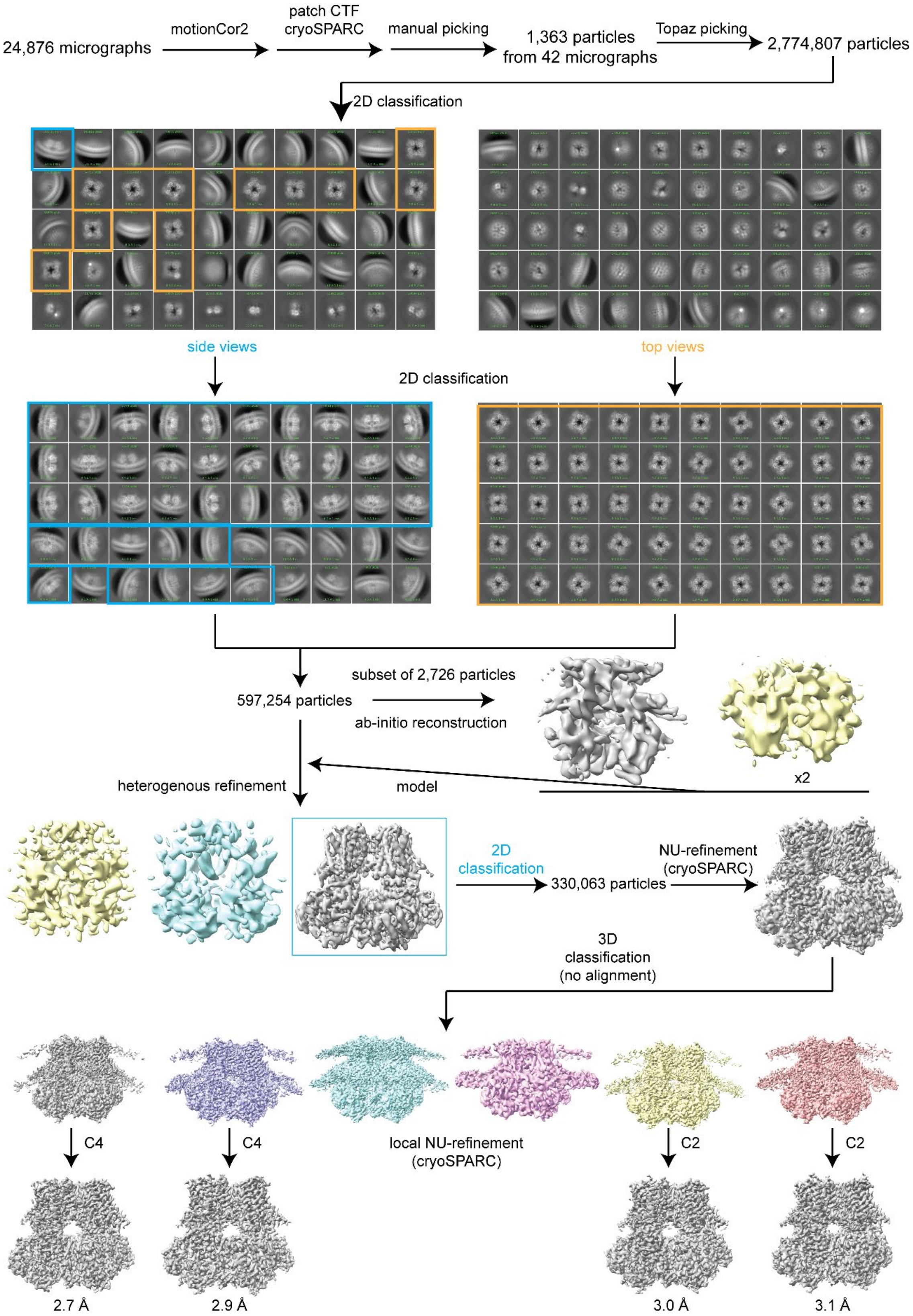

Fig. S4. Cryo-EM data processing procedure for hSlo1 in plasma membrane vesicles.

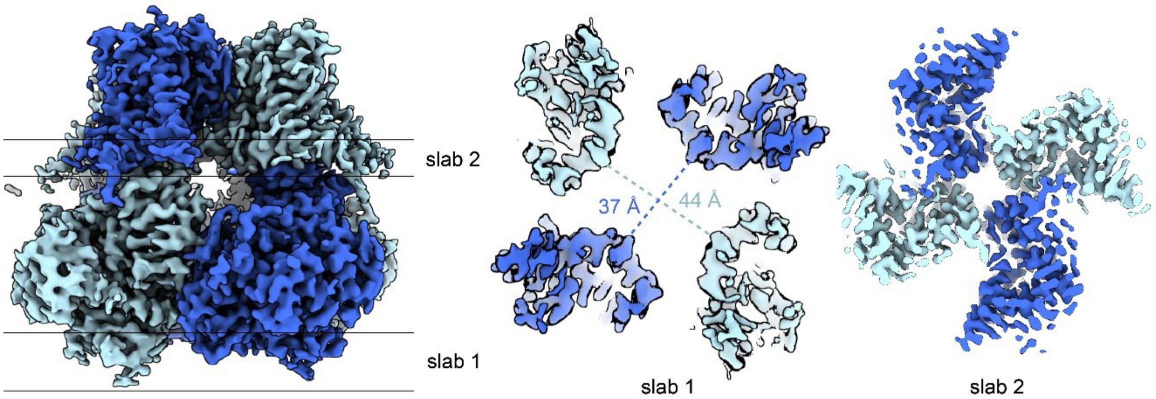

Fig. S5. 2-fold symmetrical class in hSlo1 dataset from plasma membrane vesicles. The protomers in Slo1 are colored in dark and light blue. Slabs of cryo-EM density parallel to the membrane plane are taken at the intracellular-most end of the Slo1 gating ring (slab 1) and the pore region (slab 2).

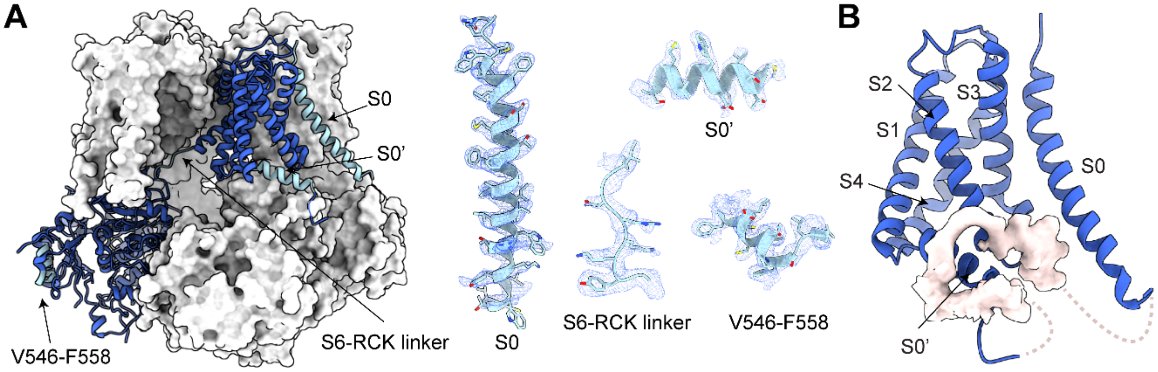

Fig. S6. Cryo-EM protein densities of exemplar regions of the Slo1 structure in plasma membrane vesicles not resolved previously. (**A**) Cryo-EM densities of S0, S0', S6-RCK linker and V546-F558 in RCK1. (**B**) Cryo-EM densities of the loop connecting S0 and S0'.

**Collection Parameters**	$hSlo1$ in total membrane vesicles (intermediate Ca^{2+}) PDB ID 8GH9 EMD-40038	$hSlo1$ in plasma membrane vesicles (2 mM Ca^{2+}) PDB ID 8GHF EMD-40044	$hSlo1$ in detergent digitonin (intermediate Ca^{2+}) PDB ID 8GHG EMD-40045
Accelerating Voltage (kV)	300	300	300
Number of frames	40 (Gatan K3)	1,505 (Falcon 4i)	40 (Gatan K3)
Dose ($e^-/Å^2$)	51.4	60	51.4
Defocus Range (µm)	-1 to -2	-1 to -2	-0.7 to -2.2
Exposure Time (s)	2	4.735	2
Original Pixel size (Å)	0.54	0.743	0.54
Spherical aberration (mm)	0.001	2.7	0.001
Amplitude contrast	0.1	0.07	0.1
**Map Parameters**			
Final Pixel Size (Å)	1.08	0.743	1.08
Symmetry	C2	C4	C2
Total micrographs	21,348	24,876	18,495
Initial particles	1,260,212	2,774,807	5,182,492
Final particles	67,716	32,063	123,286
Map resolution (Å)	3.8	2.7	3.2
FSC threshold	0.143	0.143	0.143
**Model Composition**			
Nonhydrogen atoms	27,066	31,920	26,659
Protein residues	3,389	3,640	3,535
Ligands	0	128	0
r.m.s.d. bond length (Å)	0.004	0.002	0.003
r.m.s.d. bond angle	0.750	0.594	0.696
**Validation**			
MolProbity Score	1.95	1.61	1.80
Clash Score	9.29	2.00	7.93
Poor Rotamers (%)	0.37	3.45	0.10
Ramachandran Plot			
Favored (%)	92.84	97.41	94.67
Allowed (%)	7.16	2.59	5.33
Disallowed (%)	0	0	0

Table S1. Cryo-EM data collection, map parameters and validation statistics.

Movie S1 (separate file). Protein and lipid density in the cryo-EM map of hSlo1 in plasma membrane vesicles. Protein residues are colored in white, lipid molecules in yellow, and the cryo-EM density in blue. When only the protein density is shown, the protein model is represented as sticks. When only the lipid density is shown, the protein model is rendered as a Cα trace, and the lipid molecules are represented as sticks.
